# Stereoscopic virtual reality models for planning tumor resection in the sellar region

**DOI:** 10.1186/1471-2377-12-146

**Published:** 2012-11-28

**Authors:** Shou-sen Wang, Shang-ming Zhang, Jun-jie Jing

**Affiliations:** 1 Department of Neurosurgery, Fuzhou General Hospital, Fujian Medical University, 156 Xihuanbei Road, Fuzhou, 350025, China

**Keywords:** Dextroscope, Three-dimensional reconstructionm, Transsphenoidal approach, Sellar tumor, Surgery simulation

## Abstract

**Background:**

It is difficult for neurosurgeons to perceive the complex three-dimensional anatomical relationships in the sellar region.

**Methods:**

To investigate the value of using a virtual reality system for planning resection of sellar region tumors. The study included 60 patients with sellar tumors. All patients underwent computed tomography angiography, MRI-T1W1, and contrast enhanced MRI-T1W1 image sequence scanning. The CT and MRI scanning data were collected and then imported into a Dextroscope imaging workstation, a virtual reality system that allows structures to be viewed stereoscopically. During preoperative assessment, typical images for each patient were chosen and printed out for use by the surgeons as references during surgery.

**Results:**

All sellar tumor models clearly displayed bone, the internal carotid artery, circle of Willis and its branches, the optic nerve and chiasm, ventricular system, tumor, brain, soft tissue and adjacent structures. Depending on the location of the tumors, we simulated the transmononasal sphenoid sinus approach, transpterional approach, and other approaches. Eleven surgeons who used virtual reality models completed a survey questionnaire. Nine of the participants said that the virtual reality images were superior to other images but that other images needed to be used in combination with the virtual reality images.

**Conclusions:**

The three-dimensional virtual reality models were helpful for individualized planning of surgery in the sellar region. Virtual reality appears to be promising as a valuable tool for sellar region surgery in the future.

## Background

A major challenge for neurosurgeons who perform sellar tumor operations is to perceive accurately the complex relationships of the anatomical structures. Blood is supplied to the posterior lobe of the pituitary gland and pituitary stalk by the superior and inferior hypophyseal branch of the internal carotid artery (ICA) and to the anterior lobe by the penetrating capillary loops from the portal vessels of the hypophyseal–portal circulation [1]. Aydin et al. used cadavers to describe the anatomical features and variations of the structures in this region such as the sella turcica, sphenoid ostia, sphenoid sinus and septae, optic protuberance, and carotid protuberance [2]. They also described the various supra and parasellar neurovascular structures.

The preferred treatment for pituitary adenomas and other lesions in the sellar area is microsurgery using the transsphenoidal approach [2]. As an alternative, endoscopic transsphenoidal surgery is sometimes used. Regardless of whether microsurgery or endoscopy is used, it is critical that the surgeon understands the anatomy of the sellar region. The anatomical studies on sellar tumors and surrounding structures are mainly based on cadaver specimens and two-dimensional cross-sectional images [3]. Cadaver specimens do not have individual characteristics, and cannot be used repeatedly, and therefore, they are of limited value for individual cases in clinical practice. Also, two-dimensional cross-sectional images such as those obtained from CT and MRI can barely reflect the spatial relationship of the approach-related anatomy comprehensively. Therefore, there is a need for a more suitable tool and method to provide a basis for understanding sellar region anatomy.

Virtual reality (VR) appears to be a promising technique for obtaining accurate anatomical information that is directly applicable to surgery on the brain. There have been reports on the use of virtual reality to treat various conditions including skull base tumors [4], fourth ventricular ependymoma [5], isolated orbital blowout fractures [6], microvascular decompression in the cerebellopontine angle [7], arterio-venous malformation [8,9], cerebral gliomas adjacent to motor pathways [10], and cranial nerve microvascular decompression [11]. The results in these reports have been promising.

To our knowledge, there have been no reports so far on using virtual reality for lesions in the sellar area. Therefore, the aim of this study was to investigate the value of using virtual reality for surgery to resect sellar tumors.

## Methods

### Patients

Sixty patients were randomly selected from patients admitted to the Fuzhou General Hospital from October 2009 to March 2011 because of sellar tumors and who were scheduled for surgery. Patients were excluded if they did not undergo head CT or thin slice MRI at our hospital. There were 28 male and 32 female patients; their ages ranged from 7–75 years ( average: 50.2 years). This study was approved by the IRB of Fuzhou General Hospital, Fujian Medical University and all patients signed informed consent form. Parental consent was obtained for patients under 16 years old. The types of tumors diagnosed in the 60 patients are listed in Table 1.

**Table 1:** Actual surgery data of the tumor models

**Tumor models**	**Number of cases**	**Surgical approach**	**Surgical effects**	**Complications**
Large pituitary adenoma	18	Transmononasal sphenoid sinus approach	Total resection(14)/ subtotal resection(4)	No
Giant pituitary adenoma	12	Transmononasal sphenoid sinus approach(12)	Total resection(7)/ subtotal resection(5)	No
Rathke cleft cyst	1	Transpterional approach	Total resection	No
Sphenoid ridge meningioma	10	Transpterional approach	Total resection	No
Tuberculum sellae meningioma	9	Transpterional approach(8)/ Subfrontal approach(1)	Total resection	No
Craniopharyngioma	7	Transpterional approach(4)/ Anterior interhemispheric approach(3)	Total resection(6)/ subtotal resection(1)	No
Cavernous sinus hemangioma	1	Transpterional approach	Total resection	No
Huge B-cell lymphoma in the suprasellar region	1	Transpterional approach	Total resection	No
Chordoma of the upper clivus	1	Transmononasal sphenoid sinus approach	Total resection	No

### Instruments and equipment

Image sequence scanning equipment included a 64-slice spiral CT scanner (Discovery Ultra, GE) and 3.0T MRI scanner (Trio Tim, SIE Company). The data carrier medium was a common CD-ROM with a DICOM3 format. The data processing equipment was a VR image workstation (Dextroscope, Bracco Diagnostics).

### Experimental methods

Data Acquisition: All the patients underwent thin slice CT angiography (CTA), MRI-T1WI, and contrast-enhanced MRI-T1WI image sequence scanning. Sequence requirements: the CT scan used a continuous axial scan, the slice thickness was 0.625 mm, the interval was 0 mm, and the scan time was 1.2 s. The field of view (FOV) was 250 mm × 250 mm, and the matrix was 512 × 512. The same scanning parameters were used for CTA. Iodinated contrast media was injected via the ulnar vein at a dose of 2 ml/kg body weight and a speed of 3.5 ml/s. The time window for the scan was 25 s. The MRI scan included T1WI and contrast-enhanced continuous coronal thin slice T1WI. The slice thickness was 1.0 mm, the FOV was 250 mm × 250 mm, and the matrix was 256 × 256. Scanning was continuous for 6 min. For the enhanced scan, the contrast agent gadolinium-DTPA (Gd-DTPA) was injected via the cubital vein at a dose of 0.2 mmol/kg and a speed of 3.6 ml/s. Then the contrast-enhanced scan was completed. All the image sequence data were stored in a CD-ROM in DICOM format.

Reconstruction of the structures: The sequences were duplicated, and the micro-distance and grayscale adjustment tools were used to adjust the target anatomical structures so that the soft tissue in the nasal cavity, blood vessels at the base of the skull, bone mass, the optic nerve and optic chiasm, and tumors could be displayed independently. The virtual grinding tools were used to polish and trim the anatomy, and the various structures were finally differentiated and indicated by different colors.

Simulation of the surgery using VR: The angle of view of the operating microscope was selected through changing the window size and the angle of view. Virtual instruments such as a drill and cutting tools were used to simulate surgical steps including flap fenestration, visual field exposure, and resection of the tumor. Finally, voxel editing tools were used for the rotation observation and comprehensive assessment of the extent of tumor resection. Through repeated simulations, intraoperative precautions and difficulties were identified.

All the patients underwent thin slice CT or CTA, MRI-T1WI, and contrast-enhanced MRI-T1WI image sequence scanning 1 to 3 days before surgery. Construction of each model was completed within 1 h, and preoperative simulation and evaluation were completed within 30 min. All the image sequence information was obtained within 3 days before surgery. During the preoperative assessment, typical images of each patient were selected and printed out. They were used by the surgeons as references in the operating room.

### Statistical analysis

Patient characteristic data were presented as the mean with standard deviation or frequency with percentage.

## Results

### Overall image effect of the sellar tumor model

For all models, multiple anatomical structures were constructed successfully. The integration effects between sequences were good, and the three-dimensional displays of the skin soft tissue, sellar bone mass, paranasal sinuses, optic nerve and chiasm, optic canal, ICA, circle of Willis and its branches, brain tissue, ventricular system and configuration of the tumor and its spatial relationship were clear.

### Application of the digital sellar tumor model in surgical planning

The images of the planes containing the surgical approach-related anatomical structures were extracted from the model and displayed simultaneously in the VR environment. According to the perspective of the surgical approach, the anatomical structures and key points for the operation were analyzed. Virtual instruments were used to simulate the operating process based on the actual surgical requirement for the instruments, and individualized surgical plans were developed. The surgical results showed that good operative field and exposure were obtained during the surgeries conducted according to the surgical plan, and the intraoperative findings were consistent with the results of the preoperative simulation. Thirty-one surgeries via the transmononasal sphenoid sinus approach, 25 surgeries via the transpterional approach, 3 surgeries via the interhemispheric approach, and 1 surgery via the subfrontal approach were performed. Total resection of the tumor was performed in 50 patients and subtotal resection (≥90%) was performed in 10 patients. There were no intraoperative deaths or severe postoperative complications (Table 1).

### Transmononasal sphenoid sinus approach

For the pituitary adenomas that were mainly located at the sphenoid sinus or within the sellar region, we successfully simulated surgery via the transmononasal sphenoid sinus approach. The structures, including the soft tissue in the nasal cavity, sellar bone mass, sphenoid sinus, ethmoid sinus, ICA, optic nerve, and hypothalamus, were extracted separately and were displayed in different colors. The configuration of the anatomical markers, including the nasal septum, turbinate, posterior nare, sphenoid sinus openings, sphenoidal crest, sphenoid sinus cavity, and sellar floor, were observed for intraoperative localization.

**Figure 1:**
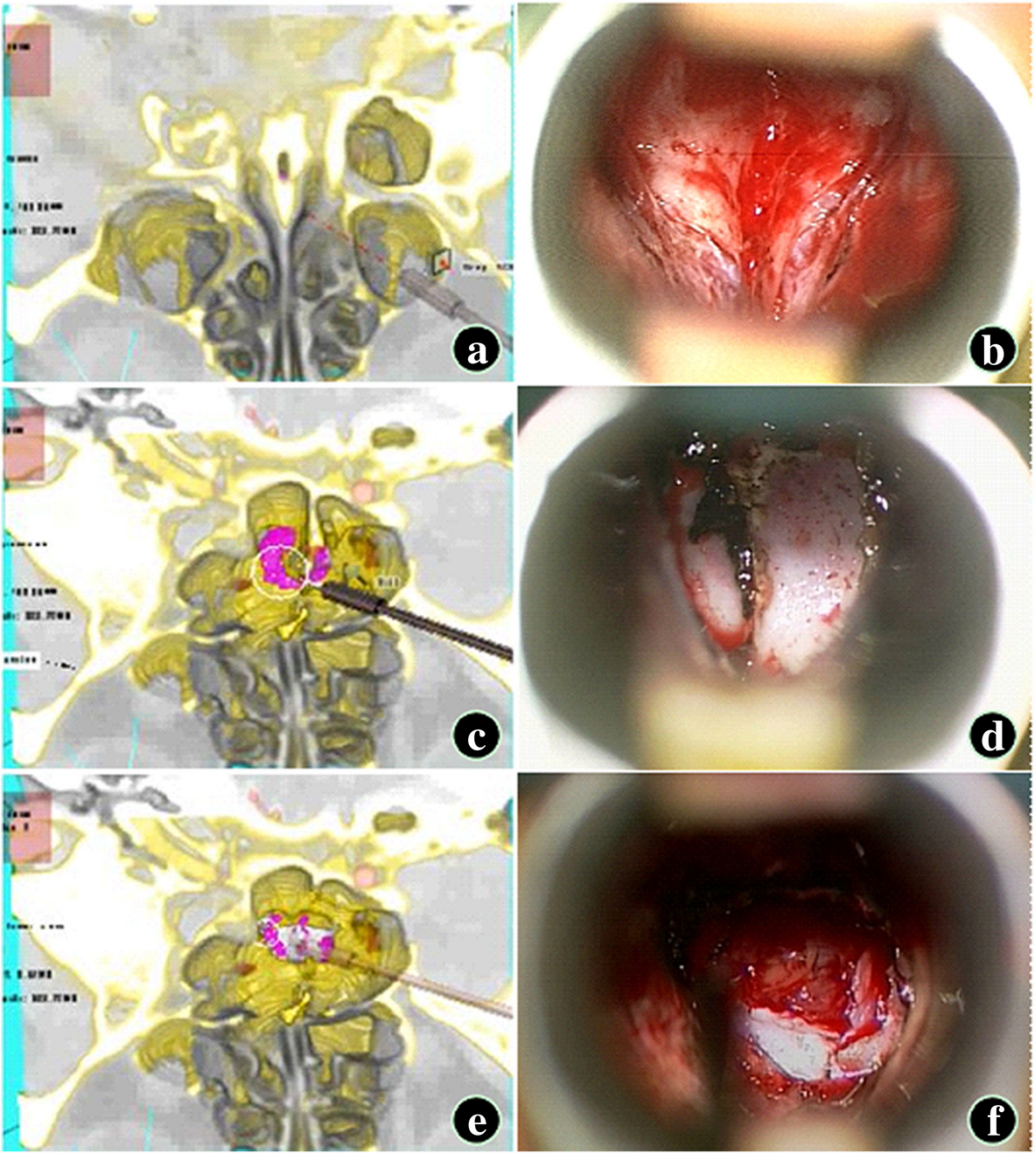
**Simulation of the transmononasal sphenoid sinus approach (large pituitary adenoma model).** (**a** and **b**) Exploration and exposure of the sphenoid sinus opening; the "boat head" sign was seen, which was consistent with the intraoperative finding. (**c** and **d**) A sphenoid sinus septum was seen in the sphenoid sinus cavity, which was consistent with the intraoperative finding. (**e** and **f**) The tumor was visible after the sellar floor was opened, and the observation angle could be adjusted to guide the extent of the sellar floor opening (VR: Pink - tumor ; Red - the internal carotid artery; Yellow - bone mass; White - the optic nerve; Brown - soft tissue ).

The surgery was completed under a microscope in all patients. The intraoperative anatomy was consistent with the results of the surgery simulation (Figure 1). It is worth noting that some fine structures were difficult to show in the models including the sphenopalatine artery, pituitary and the pituitary stalk, and dura mater on the sellar floor, as was the intraoperative shift of the structures and sinking of the tumor.

### Transpterional approach

**Figure 2:**
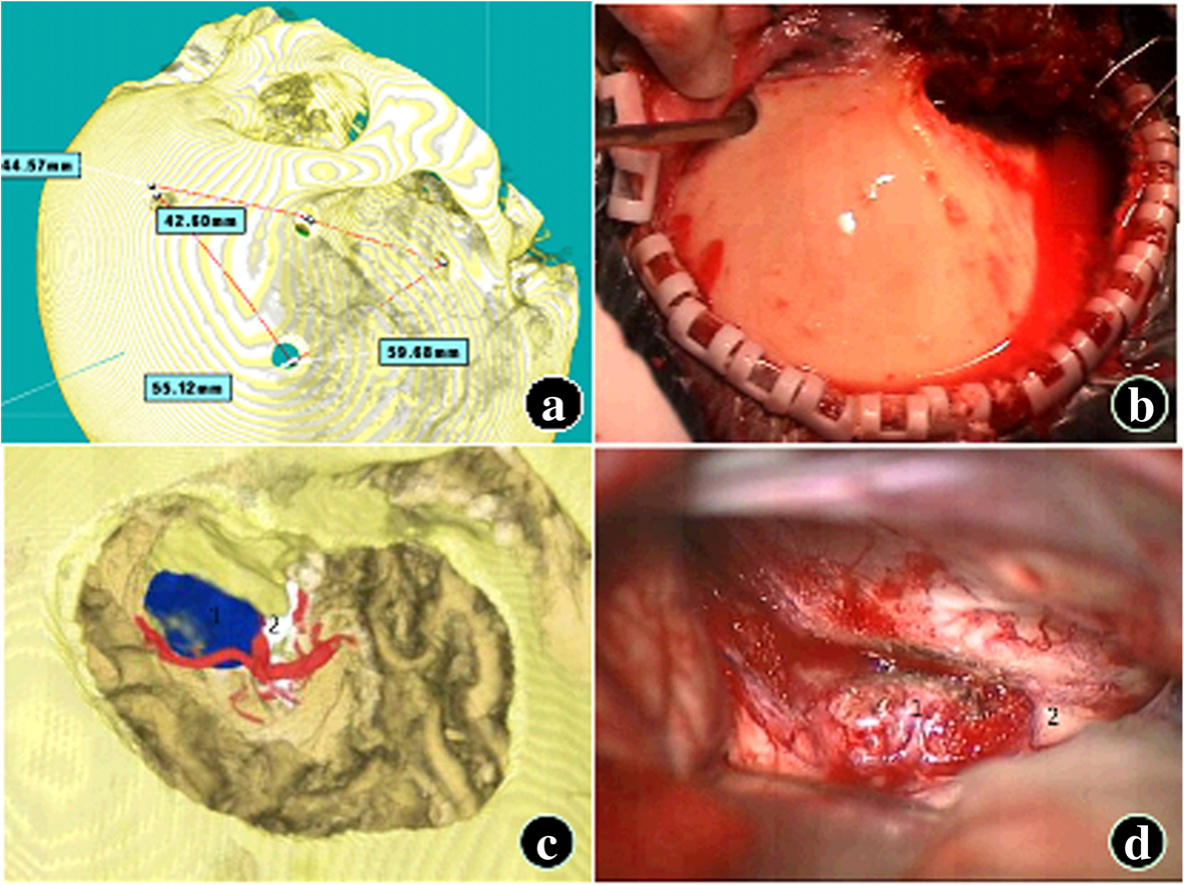
**Simulation of the transpterional approach (tuberculum sellae meningioma model).** (**a** and **b**) Development of the bone fenestration plan. (**c** and **d**) Simulation of the intraoperative traction of the frontotemporal lobe (1: Tumor; 2: Optic nerve; VR: Blue – tumor; Red – artery; Yellow - bone mass; White –optic nerve; Brown - frontotemporal lobe).

For tumors that mainly expanded toward the suprasellar, anterior sellar, parasellar, and posterior sellar regions, including giant pituitary adenoma, sphenoid ridge medial meningioma, tuberculum sellae meningioma, and craniopharyngioma, surgery via the transpterional approach was successfully simulated. The tumor growth, size, nature, and the surrounding involvement were assessed through three-dimensional rotation to determine whether the tumor wrapped around or had any adhesions with the optic chiasm, hypothalamus, and and and its branches. For the tumors that invaded the branches of the anterior and middle cerebral artery, the blood supply was assessed, and a decision was made whether preoperative embolization was necessary and what the embolization range would be. According to the observations, the size and location of the bone window was determined (Figure 2a, b) to allow full exposure of the tumor. The gaps I, II, and III and the gap above the ICA in the sellar region were measured, and the operation space and precautions were identified. The volume of the tumor part in the optic canal or at the anterior skull base was measured so that the grinding degree of the anterior clinoid process and the optic canal was determined. The rationality of each exposure approach was assessed and injury to the ICA and optic nerve was prevented.

In this group of patients, to simulate the frontal and temporal lobe traction for the tumor exposure during actual surgery, some part of the frontal and temporal lobe was grinded off; the simulation results were consistent with the intraoperative findings (Figure 2c, d). Some subtle anatomical structures such as the pituitary stalk and circle of Willis could not be shown in the model as well as the shift of tissues during the actual surgery.

### Other approaches

**Figure 3:**
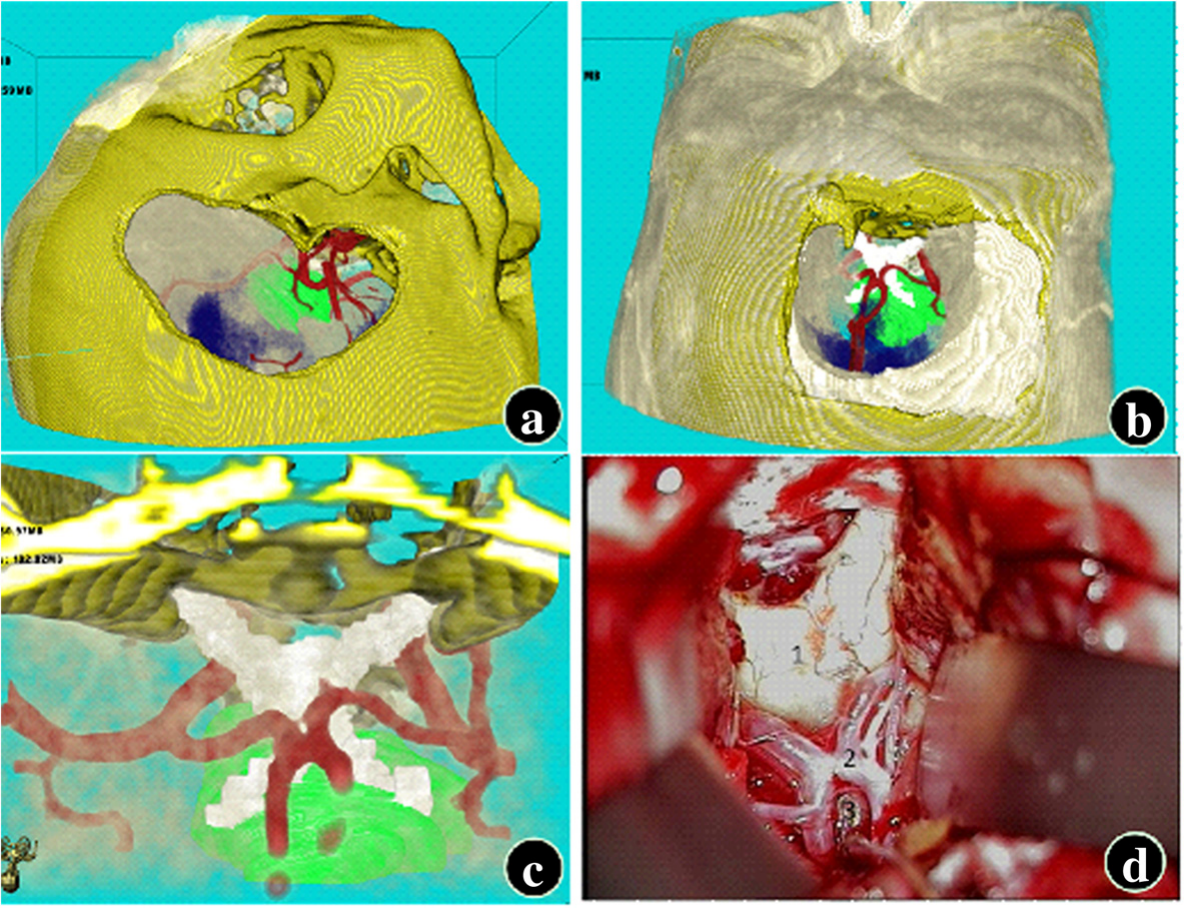
**Simulation of the anterior interhemispheric-lamina terminalis approach (craniopharyngioma model).** The structures adjacent to the tumor were observed and the advantages and disadvantages of the transpterional approach (**a**) and interhemispheric approach (**b**) were compared to determine the surgical approach. The surgical approach was selected, and the lamina terminalis was opened to expose the tumor. The simulation results (**c**) were consistent with the intraoperative findings (**d**) (1: Prefixed optic chiasm; 2: Anterior communicating artery; 3: Lamina terminalis) (VR: Blue - ventricles; Green – tumor; Red – arteries; Yellow - bone mass; White - optic nerve).

For special tumors in the suprasellar region, preconstruction of the structures including the tumors, bone mass in the sellar region, optic nerve, ICA, circle of Willis and its branches, brainstem, and ventricles could be performed. The final surgical approach could be chosen based on the observations and assessment results. For example, a craniopharyngioma was mainly located in the third ventricle in a patient, and the assessment showed that with the transpterional approach it is difficult to expost the whole tumor and easy to injure the optic chiasm, hypothalamus, and pituitary stalk. Therefore, we chose the anterior interhemispheric approach because it was reasonable and we were acquainted with it (Figure 3a, b). Structures including the vertex, anterior interhemispheric fissure, corpus callosum, bilateral ventricles, and interventricular foramen were extracted and surgery via the anterior interhemispheric approach was simulated. Actual surgery was performed according to the simulated surgical plan (Figure 3c, d).

**Figure 4:**
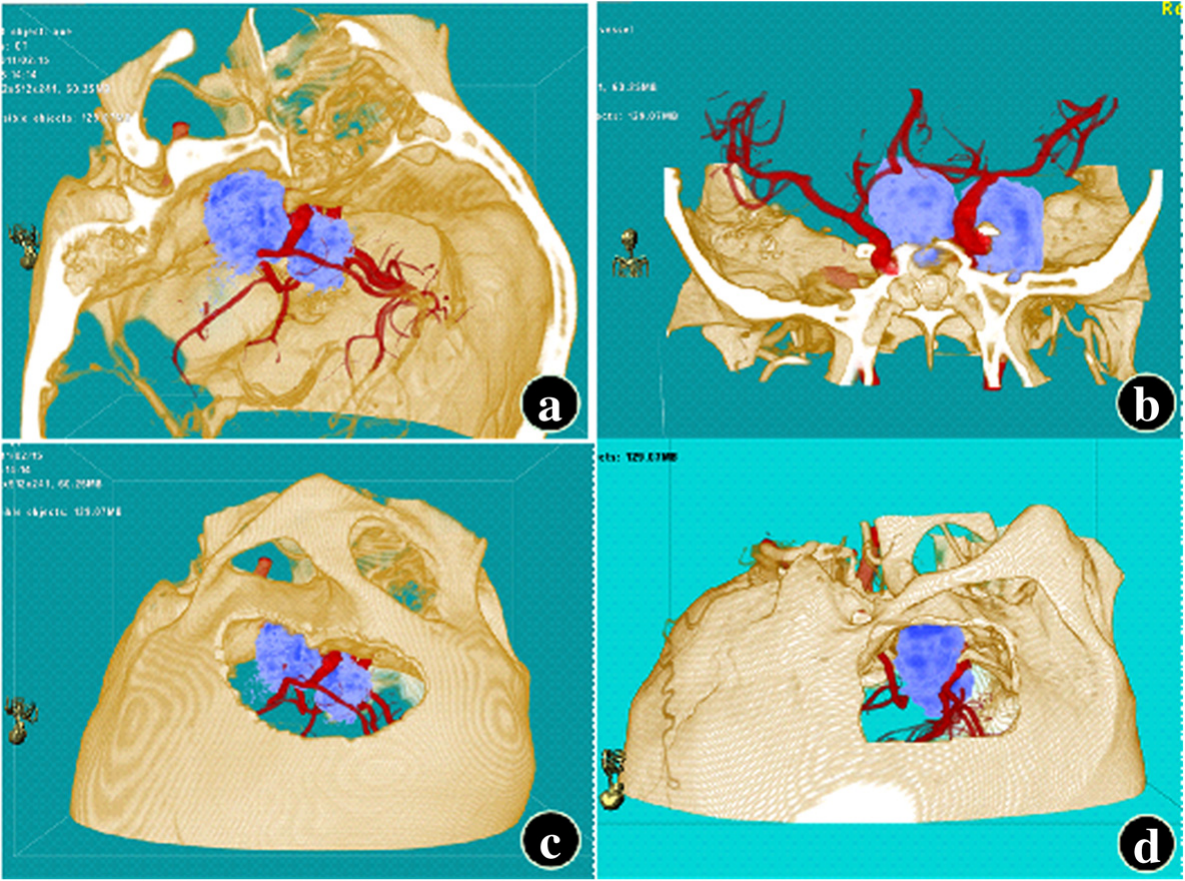
**Preoperative simulation of a model of cavernous hemangioma in the cavernous sinus.** (**a**) In the left anterior view, the tumor was closely related to the cavernous sinus. (**b**) In the coronal view, the tumor expanded toward the right suprasellar region. (**c**) Simulation of the transpterional approach. (**d**) Simulation of the subtemporal approach (Red – arteries; Yellow - bone mass; Light blue - tumor).

When the tumor model with intracavernous invasion was used to simulate the surgical approach, the display resolution of some fine structures including the dura mater in the cavernous sinus, oculomotor nerve, trochlear nerve, and trigeminal nerve was poor in two-dimensional cross-sections and they could not be reconstructed in VR. The surrounding structures of the tumor could be assessed using two-dimensional cross-sectional images to determine the surgical approach. In VR, with the in situ display of the bone mass, lobes, and brainstem, the visual field via the subtemporal approach with a lower border as low as the middle cranial fossa bottom basically could be simulated. The exposure extent of the tumor, ICA, and cavernous sinus could be assessed; however, the results of the simulation were poor. Preoperative simulation based on a model of cavernous hemangioma within the cavernous sinus (Figure 4) showed that tumor expanded toward the right suprasellar region could not be resected completly via the subtemporal approach and total resection required the transpterional approach.

Due to the tumor characteristics and the surgeon's personal preferences, the constructed models in this study were applied in surgical planning for other approaches such as the expanded transnasal approach and supraorbital keyhole approach.

The specific postoperative complications in 30 patients with pituitary adenomas were described. Changes in hormone levels and vision and visual field were improved. However, cerebrospinal fluid leakage occurred in one patient which was cured after absolute bed rest. One patient experienced diabetes insipidus, which was alleviated after administration of desmopressin and was cured at 3 months follow-up. One patient had severe diabetes insipidus, which was cured after pituitrin treatment. Transient diabetes insipidus occurred in two patients.

Thirty cases of pituitary adenoma models were researched in detail based on the sellar tumor models.

Thirty patients were randomly selected from the 60 patients. There were 14 male and 16 female patients; their ages ranged from 17–73 years (average: 47.7 years). The clinical characteristics and anatomical measurements of these patients are presented in Table 2.

**Table 2:** Clinical characteristics and anatomical measurements of 30 patients with sellar tumors

	**Mean ± SD**	**Range**
Age	47.67±13.93	17-73
Male (%)	14(46.67%)	
Hardy class (%)		
1	1(3.33%)	
2	8(26.67%)	
3	10(33.33%)	
4	5(16.67%)	
5	6(20%)	
Length of stay	18.57±6.43	10-38
Distance from the midpoint of the columella to the bone-cartilage junction of the nasal septum (mm)	29.9±1.66	26.95-34.09
Distance from the external naris plane to the most shallow point on the anterior wall of the right sphenoid sinus (mm)	62.49±4.96	51.37-71.56
Distance from the external naris plane to the most shallow point on the anterior wall of the left sphenoid sinus (mm)	62.98±5.02	52.97-71.99
Distance from the sphenoid sinus opening to the midpoint of the columella (mm)	66.14±5.2	55.22-75.97
Distance from the sphenoid sinus opening to the choanal upper edge (mm)	26.66±2.1	23.17-29.92
Distance from the sphenoid sinus opening to the hanging wall (mm)	16.83±3.5	11.29-25.6
Distance from the sphenoid sinus opening to the inferior wall (mm)	10.47±3.23	6.16-19.87
Distance from the sphenoid sinus opening to the right lateral wall (mm)	18.26±3.94	12.74-25.41
Distance from the sphenoid sinus opening to the left lateral wall (mm)	18.13±3.94	12.53-25.97
Angle between the connecting line from the columella to the glabella and the approach (°)	54.54±2.48	49.81-60.02
Distance from the columella - glabella extension line to the sellar floor (mm)	15.97±7.23	1.39-29.3

### Survey Questionnaire

Eleven surgeons who used this VR tool completed a survey questionnaire. Their perceptions on the relationship between the model and actual surgery were as follows: no difference (n=0); some differences are present, but the model still helps with understanding the anatomy (n=10); differences are significant and the model is not conducive to observing the anatomical structures (n=1); no similarities (n=0). Their perceptions about the advantages of the model compared with conventional two-dimensional images that benefit surgery were the following: no other images are needed (n=0); superior to other images, but the use of other images needs to be combined (n=9); same as other images, the model is optional (n=2); the model was of no help (n=0).

## Discussion

Our results indicate that VR is helpful for planning sellar area surgery and performing operations in the sellar area. Among 11 surgeons who used VR and completed a survey questionnaire, 10 surgeons perceived that there were some differences between the model and the actual anatomy observed during surgery, but that the model does help with understanding the anatomy, and 9 surgeons thought the VR images were superior to two-dimensional images but there was still a need for other images to be used in combination with the VR images.

To the best of our knowledge, there has been only one prospective controlled study in which patients who were operated on after VR was used for preoperative planning were compared with patients who were operated on without VR being used for preoperative planning [4]. All of the patients had skull base tumors. The patients for whom VR was used had significantly shorter operation duration and length of stay, and significantly more improvement in Karnofsky performance scores at discharge and at 6 months follow-up. The VR group also had significantly fewer cerebrovascular injury complications. These results suggest that the use of VR can improve outcome in patients with skull base tumors. Future research is needed to determine whether using VR can improve outcome for patients with sellar tumors.

Traditional surgery for sellar tumors often relies on two-dimensional cross-sectional images and three-dimensional thinking ability of experienced surgeons for the surgical planning. Due to limited abstract ability of individuals and bias, surgeons may have different understanding of the lesions, which may affect the precise implementation of surgery. The surgical planning system based on VR allows multi-angle, multi-dimensional observation of the morphology of tumors and adjacent structures, and approach-related anatomical information to formulate a precise surgical plan. Therefore, it may increase the confidence of the surgeons and improve the visualization of anatomical structures during surgery [12-14].

The digital model of sellar tumors is based on the VR technique. The simulated surgical approach by the sellar tumor model can be used as reference for the actual surgery, which can overcome the shortcomings of conventional two-dimensional images. Compared with (such as spiral CT images), it is easy to observe the various anatomical structures in the surgical approach and the spatial relationship between the tumor and the adjacent structures in the three-dimensional digital model. The tumor characteristics and anatomical shift in individual cases cannot be shown by anatomical mapping and a surgical atlas. Therefore, the main advantage of the model application is the individualized characteristics. Neuronavigation systems can provide reliable actual anatomical information for surgeons; if the use of VR technique and neuronavigation systems can be combined, this may provide more helpful guidance for surgery [15].

The anatomical structures displayed by digital sellar tumor models are realistic and clear, and a variety of virtual tools can be used to simulate the actual anatomical tools [16] .Therefore, they can be used to complement cadaveric head specimens. They may also complement the conventional surgical planning library and teaching library, and provide an effective basis for clinical surgery. However, the constructed models in this study were not based on a large number of cases, and we still could not establish an evidence-based database that is large enough.

The models constructed in this study are not perfect and have many shortcomings. For example, they are limited by the resolution and slice thickness of current MRI and CT technology. They cannot display the fine structures in the actual anatomy including the dura mater, the majority of cranial nerves, and the majority of small blood vessels. The display of the three-dimensional configuration relies on the use of specific techniques, and they cannot be performed in the operating room. Virtual reality cannot truly simulate the displacement of tissue structures during the actual surgery and the texture of the entity so that it cannot be a real-time tool to guide the surgical process. The development of a more clear and thin-slice image sequence and a powerful computational processing system would help improve the display of fine structures. Combined with other techniques including intraoperative MRI, virtual endoscopy, neuronavigation systems, and digital physical feedback, the model may be endowed with intraoperative real-time marking and analog feedback function [17,18].

However, our study had several limitations: 1) The quality of the reconstructed images varied according to the resolution of the existing MRI and CT technology and by slice thickness. Also, demonstrating the fine intra-operative microstructures was difficult. Therefore, this technology could not completely replace autopsy, which is the gold standard. 2) This technology could only be used in hospitals that possess the appropriate hardware. 3) Regarding the actual displacement and texture of the tissue structures during surgery, this technology cannot genuinely simulate and provide feedback, confirm the accuracy of the locations of various structures, or reflect the condition of structural resection/displacement. Hence, real-time operation guidance is lacking. 4) The reconstructed models and images focused on pre-operative planning, which is beneficial for surgeons who utilize a three-dimensional model but not for surgeons who are used to routine cross-sectional images for pre-operative planning.

## Conclusions

Virtual reality provides three-dimensional anatomical models that appear to be useful for individualized pre-operative planning for surgery in the sellar region.

## Competing interest

The authors declared that they have no competing interest.

## Authors’ contributions

SW carried out the study design, participated in the clinical studies and drafted the manuscript. SZ carried out the experimental studies, data acquisition, data analysis and statistical analysis. JJ participated in literature research, clinical studies, manuscript preparation and editing. All authors read and approved the final manuscript.

## Pre-publication history

The pre-publication history for this paper can be accessed here:

http://www.biomedcentral.com/1471-2377/12/146/prepub
